# Injected human umbilical cord-derived mesenchymal stromal cells do not appear to elicit an inflammatory response in a murine model of osteoarthritis

**DOI:** 10.1016/j.ocarto.2020.100044

**Published:** 2020-06

**Authors:** J. Perry, H.S. McCarthy, G. Bou-Gharios, R. van 't Hof, P.I. Milner, C. Mennan, S. Roberts

**Affiliations:** a Robert Jones & Agnes Hunt Orthopaedic Hospital NHS Foundation Trust, Oswestry, SY10 7AG, UK; b School of Pharmacy and Bioengineering (PhaB), Keele University, Keele, ST4 7QB, UK; c Institute of Ageing and Chronic Disease, University of Liverpool, L7 8TX, UK

**Keywords:** Allogeneic cell therapy, Human MSCs, OA, Preclinical model, Xenogeneic

## Abstract

**Objective:**

This study investigated the effect of hUC-MSCs on osteoarthritis (OA) progression in a xenogeneic model.

**Design:**

Male, 10 week-old C57BL/6 mice underwent sham surgery (n = 15) or partial medial meniscectomy (PMM; n = 76). 5x10^5^ hUC-MSCs (from 3 donors: D1, D2 and D3) were phenotyped via RT-qPCR and immunoprofiling their response to inflammatory stimuli.

They were injected into the mouse joints 3 and 6 weeks post-surgery, harvesting joints at 8 and 12 weeks post-surgery, respectively. A no cell ‘control’ group was also used (n = 29). All knee joints were assessed via micro-computed tomography (μCT) and histology and 10 plasma markers were analysed at 12 weeks.

**Results:**

PMM resulted in cartilage loss and osteophyte formation resembling human OA at both time-points. Injection of one donor's hUC-MSCs into the joint significantly reduced the loss of joint space at 12 weeks post-operatively compared with the PMM control.

This ‘effective’ population of MSCs up-regulated the genes, IDO and TSG6, when stimulated with inflammatory cytokines, more than those from the other two donors.

No evidence of an inflammatory response to the injected cells in any animals, either histologically or with plasma biomarkers, arose.

**Conclusion:**

Beneficial change in a PMM joint was seen with only one hUC-MSC population, perhaps indicating that cell therapy is not appropriate for severely osteoarthritic joints. However, none of the implanted cells appeared to elicit an inflammatory response at the time-points studied. The variability of UC donors suggests some populations may be more therapeutic than others and donor characterisation is essential in developing allogeneic cell therapies.

## 1 Introduction

Osteoarthritis (OA) is not only the most common musculoskeletal disorder in our society but is also seriously debilitating, impacting more on an individual's quality of life than cancer, diabetes or heart disease [1]. Despite this, the most frequent treatment option for patients is arthroplasty at the end stage of the disease, replacing the degenerate joint with an inert prosthetic device, which has a finite lifespan. With an increasingly aged society, more arthroplasties are requiring revision and replacement year on year; these are more challenging, more expensive and less successful than the original surgery [2]. A biological approach could provide an earlier and more permanent solution.

Autologous chondrocyte implantation (ACI) has been used for over two decades for treating more discrete chondral or osteochondral defects in the knee [3,4], which if left untreated often progress to end stage osteoarthritis [5]. Although ACI has recently been reported to be cost-effective [6], an autologous cell therapy product is much more restrictive in terms of production costs, logistics and donor site morbidity than an allogeneic cell therapy product. Hence, development of an allogeneic treatment could have many advantages, with cells from tissues earlier in development for example having potential advantages over those obtained from more mature individuals [7]. We have previously characterised mesenchymal stromal cells (MSCs) isolated from whole human umbilical cord (UC) and shown them to have a greater proliferative capacity and equivalent immunomodulatory ability (in terms of gene expression of indoleamine 2,3-dioxygenase (*IDO*) and human leukocyte antigen (HLA)-G than MSCs derived from adult human bone marrow [8,9].

Studying the aetiopathogenesis of OA in humans remains a challenge, as there is restricted availability of diseased tissues particularly at the early stage of OA. As a result of this, animal models remain an increasingly popular choice for basic science studies to identify the underlying molecular mechanisms of OA, along with studying pharmacological interventions longitudinally. While spontaneous OA models exist (e.g. in mice, guinea pigs and dogs) [[10], [11], [12]], surgical models can provide numerous advantages, including reduced variability, reduced reliance on genotype and a faster onset of disease. This generally results in shorter study durations and therefore lower husbandry costs. The partial medial meniscectomy (PMM) and medial collateral ligament transection (MCLT) models were the first surgical instability models described in the mouse [13]. In C57BL/6 mice, the PMM model has been shown to produce mild degenerative changes by 2–4 weeks post-surgery, progressing to more severe degenerative changes by 8 weeks [14,15], mimicking the pathogenesis of traumatic OA in humans, rapidly resulting in end-stage OA.

In the present study, we evaluate the effect of an intra-articular injection of human UC-MSCs on the severity of disease and inflammatory response in the PMM murine model of end-stage OA. Individual MSC populations were obtained from three humans and characterised via their immunoprofile and response to inflammatory mediators to study differences between donors.

## 2 Materials and methods

### 2.1 Human umbilical cord MSC culture

Umbilical cords (n = 3; donors D1, D2 and D3) were obtained within 24 h of natural delivery following informed patient consent (ethical approval: 10/H10130/62), as previously described [8]. The human cord donors were aged 24, 31 and 33 years at the time of harvest and all had a healthy BMI (19.7–23.2). Cells were obtained from 2 to 3 cm of UC digested with type 1 collagenase (1 mg/mL, > 125 digesting units/mg wet weight; Sigma, UK) for 1 h at 37°C, before seeding into tissue culture flasks at 5×10^3^/cm^2^ and culturing in Dulbecco's Modified Eagle's Medium (DMEM/F12, Gibco, UK) containing 1% (v/v) penicillin and streptomycin (P/S, Life Sciences, UK) and 10% (v/v) foetal calf serum (FCS; Life Sciences, UK). Cells cultured were maintained in a humidified environment at 5% CO_2_, changing media every 2–3 days and culturing cells to passage (P) 3.

### 2.2 Immunoprofiling of UC-MSCs

The immunoprofile of the UC cell populations at P3 (n = 3) was assessed using flow cytometry, as previously described [8]. Unless otherwise stated all antibodies were purchased from Becton Dickinson & company (BD), Oxford, UK. In brief, cells were stained with the following antibodies against CD14, CD19, CD34, CD45, HLA-DR, CD73, CD90, CD105 [16,17]. Cells were also assessed for other markers indicative of MSC-related behaviour (CD271, Receptor Tyrosine Kinase-like Orphan Receptor 2 (ROR2) and Fibroblastic Growth Factor Receptor 3 (FGFR3), all R&D systems, Abingdon, UK), putative chondrogenic markers (CD151, CD39, CD44, CD49c, CD163, CD166) and immunomodulatory markers, CD106 and CD317 (eBioscience UK) [[18], [19], [20]]. Co-stimulatory markers CD40, CD80, CD86 were also assessed, before and after stimulation with inflammatory cytokines, as well as HLA-DR, CD39, CD73, CD106 and CD317 as these markers are known to change upon inflammatory stimulus [21]. Appropriate isotype-matched IgG controls were used throughout, analysing approximately 100,000 cells for each antibody using a FACSCanto II flow cytometer and FACS DIVA 7 software (BD).

### 2.3 Reverse transcriptase-quantitative polymerase chain reaction (RT-PCR) of UC-MSCs’ inflammatory response

hUC-MSCs were exposed to either 25 ng/mL interferon gamma (IFN-γ) or an “inflammatory cocktail” (containing 25 ng/mL IFN-γ, 10 ng/mL interleukin (IL)-1β and 50 ng/mL tumour necrosis factor (TNF)-α) for 24 h prior to harvesting.

RNA was extracted, converted to cDNA and the expression of *IDO* and TNF-stimulating gene *(TSG)-6* was assessed as previously described [9]. The relative fold change in expression for *IDO* and *TSG6* following stimulation was determined using the comparative C_T_ method [22].

### 2.4 Animals

Male, 30 g, ten-week-old C57BL/6 wild type mice ((n = 91) Harlan Laboratories, UK), were randomly assigned to either control or experimental groups, and group housed (4 mice per cage) at the University of Liverpool in a climate controlled room in ventilated polypropylene cages, with 12 h light/dark cycles and provided with *ad libitum* water and food. Animals were culled at 18–22 weeks of age. All experimental procedures complied with the 1986 Animals (Scientific Procedures) Act and the ARRIVE guidelines [23]. The University of Liverpool Animal Welfare Committee approved the animal usage and protocols used throughout the study under Home office Licence PPL70/9047.

### 2.5 Induction of OA

The modified PMM model was performed similar to that previously described, and the timings organised so that the joints were harvested at the usual endpoints of 8 and 12 weeks [14,24]. Mice were anaesthetised via inhalation of isoflurane and oxygen under aseptic conditions. A small (3–5 mm) medial para-patellar skin incision was made in the left hind limb with a number 11 scalpel blade. The anterior horn of the medial meniscus was released from the tibial plateau through transection of the medial meniscotibial ligament (MMTL). This displacement was confirmed with forceps and the incision closed (muscle/fascia to medial edge of the patellar ligament) with synthetic absorbable sutures (8-0 polyglactin 910 (Surgicryl)) before suturing intra-dermally (again with 8-0 polyglactin 910) to close the skin. Sham operated mice were treated the same, but the meniscus was left intact after identification. Following the procedure all mice were administered pain relief (buprenorphine 0.1 mg/kg intramuscular injection) and antimicrobials (enrofloxacin 5 mg/kg subcutaneous injection).

### 2.6 Cell application

**Fig. 1:**
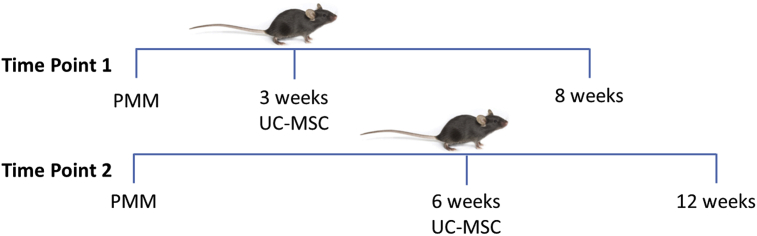
Experimental design: hUC-MSCs were injected into the hind left knee of C57BL/6 mice at either 3 or 6 weeks post-PMM (time points 1 and 2, respectively); mice were culled at 8 and 12 weeks post-PMM for time points 1 and 2 respectively. Sham operated mice and PMM control mice were also included, with neither of these receiving hUC-MSCs.

Mice were monitored for 3 weeks post-surgery for behavioural changes or poor wound healing. At 3 or 6 weeks, hUC-MSCs (5x10^5^ cells in 10 μl DMEM/F12) derived from three patient donors (D1, D2 and D3, as previously described) were injected intra-articularly to the PMM treatment groups; no cells were administered in the sham or PMM control groups (Fig. 1 & Table 1). All injections were performed under anaesthesia via inhalation of isoflurane and oxygen.

**Table 1:** Treatment groups at 8 and 12 weeks.

Group	8 weeks		12 weeks	
	PMM	SHAM	PMM	SHAM
D1 hUC-MSCs	8	–	6	–
D2 hUC-MSCs	6	–	7	–
D3 hUC-MSCs	8	–	12	–
No Cell Controls	11	11^a^	18^a^	4
Totals	33	11	43	4
	**n = 44**		**n = 47**	

D = donor.

a one mouse died in each of these groups.

### 2.7 Sample preparation

Following anaesthesia via inhalation of isoflurane and oxygen, mice were sacrificed by cervical dislocation and blood was collected (at the 12 week time-point only) via cardiac puncture and the plasma stored at −80°C. Left hind limbs of all mice were dissected, fixed in formalin (4% v/v) overnight, stored in 70% ethanol and scanned using a Skyscan 1272, μCT scanner (Bruker, Belgium), as previously described [25]. Scans were reconstructed using NRecon (Skyscan, Bruker, Belgium) and 3D volumes of interest (VOI) were identified using Dataviewer (Skyscan) and analysed using CTAn software (Skyscan). Morphometric parameters were analysed for the tibial subchondral bone epiphysis (medial/lateral Bone Volume (BV, μm [3]), Bone Volume/Tissue Volume (BV/TV as a %), subchondral bone thickness (Tb.Th, μm), total joint space (μm), osteophytes (both number and volume (μm^3^)) and total bone volume (μm^3^)), as previously described [25,26]. After μCT scanning, the knee joint specimens were returned to formalin overnight, decalcified in 200 mM EDTA for 2 weeks before transferring to 70% ethanol. Fully extended knee joints were then embedded in paraffin wax and coronally sectioned (5 μm).

### 2.8 Assessment of cartilage degeneration and synovitis

All histology was anonymised and scored by three blinded observers. Every tenth knee joint section was stained with safranin-O and fast green and scored using the Osteoarthritis Research Society International (OARSI) score [27]. In each section, four quadrants of the joint (medial femoral condyle (MFC), lateral femoral condyle (LFC), medial tibial plateau (MTP) and lateral tibial plateau (LTP)) were scored on a semi-quantitative scale from 0 to 6 (0 representing a healthy joint and 6 a severely degenerate joint). A minimum of 8 sections were scored per mouse and the highest three scoring sections throughout the knee joint were summed and averaged between scorers, to yield a maximum joint score of 72 [27].

Synovitis was evaluated on a single haematoxylin and eosin (H&E)-stained central section of the joint and assessed across all four quadrants using a modified version of a previously established scoring system [28]. A score for sub synovial stroma was included [29] whilst bone erosion was excluded (Supplementary Table 1). An immunohistochemical study was also performed on paraffin embedded sections, staining with anti-human emerin (antibodies kindly provided by Dr Heidi Fuller, Oswestry/Keele, see Supplementary data), to determine the presence of hUC-derived MSCs. (A positive control was produced by injecting 100,000 hUC-MSCs in 10 μL of medium into a mouse joint which was immediately sacrificed).

### 2.9 Biomarker analysis

Conditioned media from hUC-MSC cell cultures, of both stimulated (with an inflammatory cocktail and IFN- γ) and unstimulated cells, were analysed in triplicate for human GM-CSF, IL-1RA, IL-4, IL-6, IL-8, IL-10, IP-10, MCP-1, VEGF, SDF-1α using a custom-designed panel from MesoScale Discovery (MSD; Gaithersburg, MD, USA; see Supplementary Table 2). Mouse plasma samples from the 12 week time-point were assayed in duplicate for murine GM-CSF, IL-1β, IL-4, IL-6, IL-10, TNF-α, IFN-γ, MCP-1, VEGF and TGF-β3. All MSD plates were carried out according to the manufacturer's instructions and were read using the MSD Sector Imager 2400 and analysed with MSD Discovery Workbench software version 3.

### 2.10 Statistical analysis

All data were analysed using GraphPad Prism 8 (San Diego, USA) and tested for normality prior to analysis. Results are expressed as the mean ± standard deviation. Parametric data was analysed by one-way repeated-measures analysis of variance (ANOVA) with multiple comparisons using Tukey's correction. Non-parametric data was analysed using a Kruskal-Wallis test with Dunn's post-analysis correction with multiple comparisons. Significance was determined as p < 0.05.

## 3 Results

### 3.1 Immunoprofiling and immunomodulatory gene expression of hUC-MSCs

**Fig. 2:**
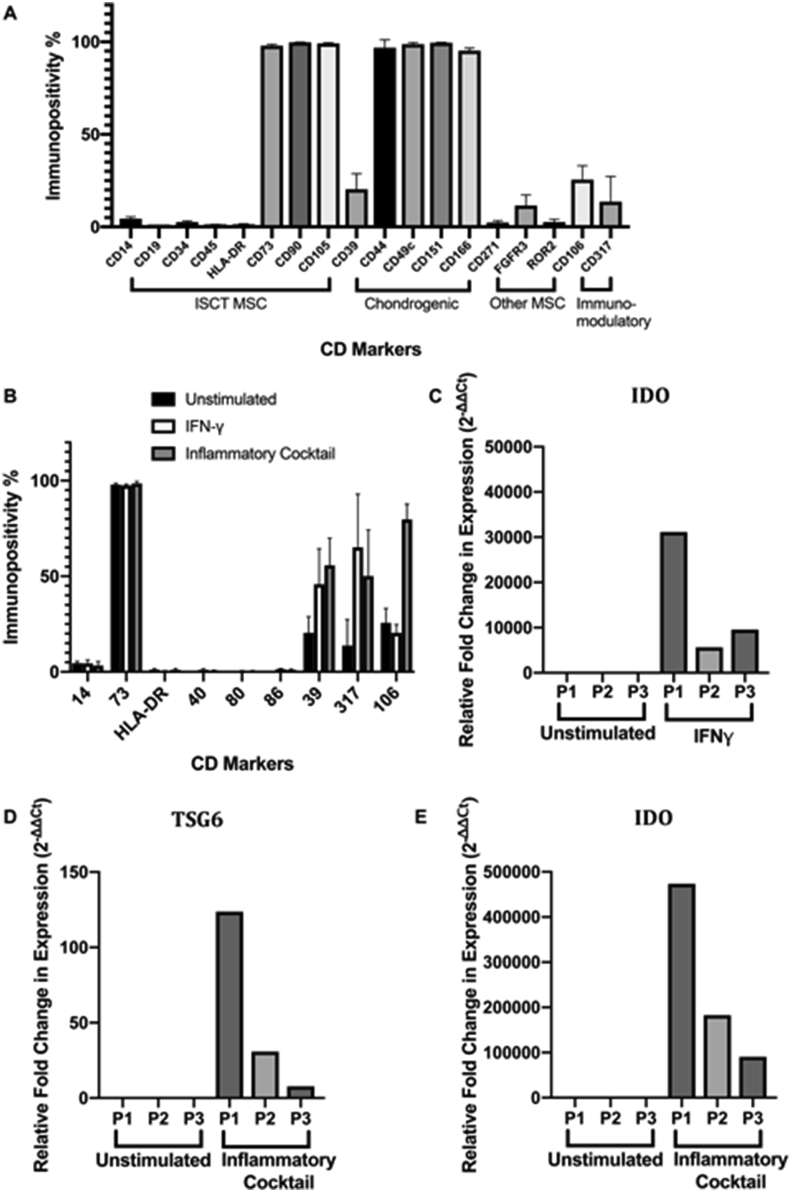
Characterisation of hUC-MSCs prior to use in the PMM model. Immunoprofiling of hUC-MSCs assessed via flow cytometry for markers indicative of MSCs, putative chondrogenic markers and immunomodulation on un-stimulated cells (A). Markers indicative of MSCs (CD14, CD73), co-stimulation (CD40, CD80, CD86), Human Leukocyte Antigen-DR (HLA-DR) and immunomodulation (CD106, CD317) on hUC-MSCs cultured in normal media without IFN-γ (unstimulated), following stimulation with 25 ng/ml IFN-γ for 24 h and following stimulation with 25 ng/ml IFN-γ, 50 ng/ml TNF-α 10 ng/ml IL-1β for 24 h (inflammatory cocktail; B). RT-qPCR analysis showing gene expression in stimulated and un-stimulated hUC-MSCs for TSG6 (stimulated with inflammatory cocktail for 24 h; C), IDO (stimulated with IFN- γ for 24 h; D) and IDO (stimulated with inflammatory cocktail for 24 h; E). Data is presented as the mean ± SD. Results are shown from 3 patients' hUC-MSCs, (D1-3).

All 3 donors had similar profiles for markers indicative of MSCs, immunomodulation and chondrogenesis. However, the hUC-MSCs did not strictly adhere to the ISCT criteria for MSCs, as CD14 was slightly elevated (mean 4.4 ± 0.97% production; Fig. 2A). Importantly, following stimulation with an inflammatory cocktail, there was no detectable production of HLA-DR or the co-stimulatory markers (CD40, CD80 and CD86), the presence of which may elicit an immune response *in vivo*. Production of the immunomodulatory markers CD39, CD106 and CD317 were increased on hUC-MSCs following inflammatory stimulus, whereas immunopositivity for CD14 and CD73 remained unchanged (Fig. 2B). *IDO* expression (Fig. 2C and E) was upregulated in all hUC-MSCs donors, to varying degrees, following stimulation with IFNγ or the inflammatory cocktail. Similar results were found for *TSG6* expression (Fig. 2D) with the inflammatory cocktail, apparently independent of age or BMI of the donor. hUC-MSCs from D1 elicited the greatest response to IFNγ and the inflammatory cocktail compared to the other 2 donors, with regards to *IDO* and *TSG6* expression (Fig. 2C–E).

### 3.2 Joint space narrowing, osteophyte formation and subchondral bone changes on μCT post-PMM surgery

**Fig. 3:**
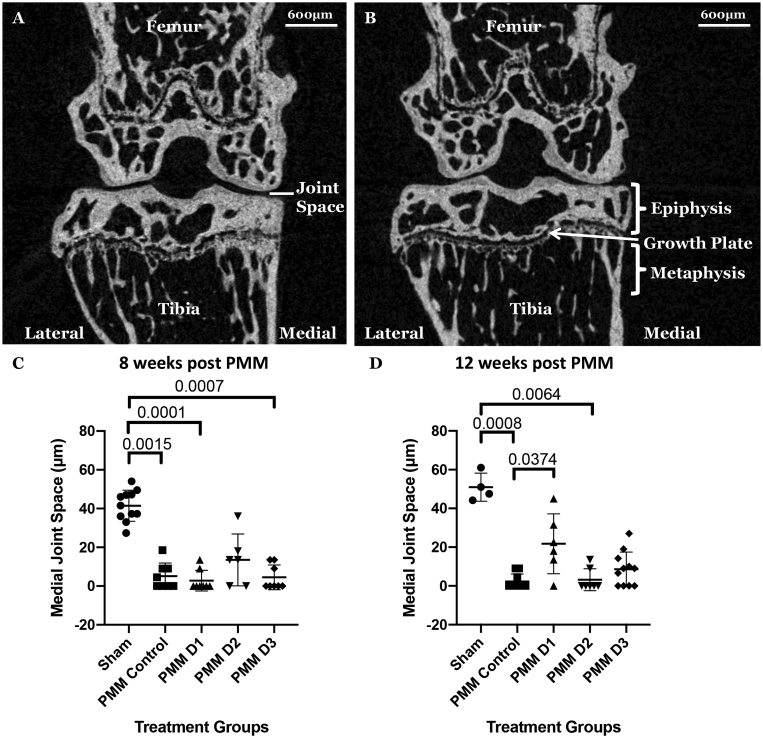
Assessment of joint space. Representative two-dimensional coronal μ-CT images at 12 weeks in sham (A) and PMM control group (B). The medial joint space of the different groups analysed by μ-CT at 8 weeks (C) indicated that all PMM groups, other than those injected with hUC-MSCs from donor D2 had a significantly reduced joint space (μm) compared to the sham control; in contrast, at 12 weeks (D) the mice injected with hUC-MSCs from donor D1 had a significantly greater joint space than the PMM control, but it was not significantly reduced compared to the sham control. Data is presented as the mean ± SD. Significance was determined below p < 0.05.

Virtual sections in the coronal plane of the three-dimensional reconstructions of the joints obtained from μCT analyses are shown in Fig. 3A and B. Micro-CT analysis revealed osteoarthritic changes in all PMM joints, with joint space narrowing and osteophyte formation evident at 8 weeks post-PMM and loss of joint space becoming progressively worse at 12 weeks after surgery. The injection of hUC-MSCs from the 3 cord donors resulted in variable outcomes in the treated mice. The mean joint space following PMM on the medial side in the PMM control group was significantly less than sham operated knees at both 8 weeks (PMM = 5.1 ± 6.7 μm, sham = 41.5 ± 8.0 μm; p = 0.0015; Fig. 3C) and 12 weeks (PMM = 2.3 ± 3.8 μm; sham = 50.9 ± 7.2 μm; p = 0.0008; Fig. 3D). At 8 weeks, cell-treated PMM groups had a similar joint space to the PMM controls but at 12 weeks there was a significantly larger joint space in mice treated with cells from D1 compared to PMM control (p = 0.0374), but not with cells from D2 or D3. The D1 joint space was also not significantly different to the sham group. On the lateral side of the joints at both the 8 and 12 week time-points, no significant differences were observed between any of the groups.

**Fig. 4:**
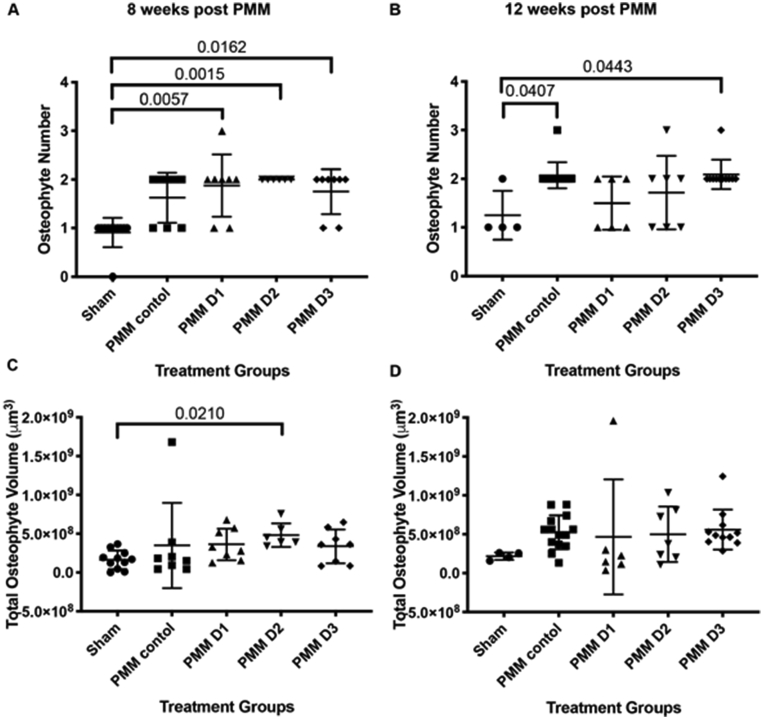
Quantification of osteophyte development. There were significantly more osteophytes in all cell groups at 8 weeks (A) but only D3 at 12 weeks (B). Total osteophyte volume was only significantly increased in animals that received hUC-MSCs from donor D2 at 8 weeks (C); there were no significant differences in any group at 12 weeks (D). Data is presented as the mean ± SD. Significance was determined below p < 0.05.

There were significantly more osteophytes in all PMM joints (Fig. 4) than sham operated knees at 8 weeks, whilst at 12 weeks, only joints that received hUC-MSCs from donor D3 and the PMM control had significantly more osteophytes than the sham control (p = 0.0443 and p = 0.0407, respectively, Fig. 4B). Mice receiving hUC-MSCs from donors D1 and D2 had fewer osteophytes than the PMM controls, but this was not significant. Furthermore, only mice that received hUC-MSCs from donor D2 at 8 weeks (p = 0.0210; Fig. 4C) had an increased total osteophyte volume compared with the sham, this difference was not seen with other PMM operated groups at either time point (Fig. 4C and D).

**Fig. 5:**
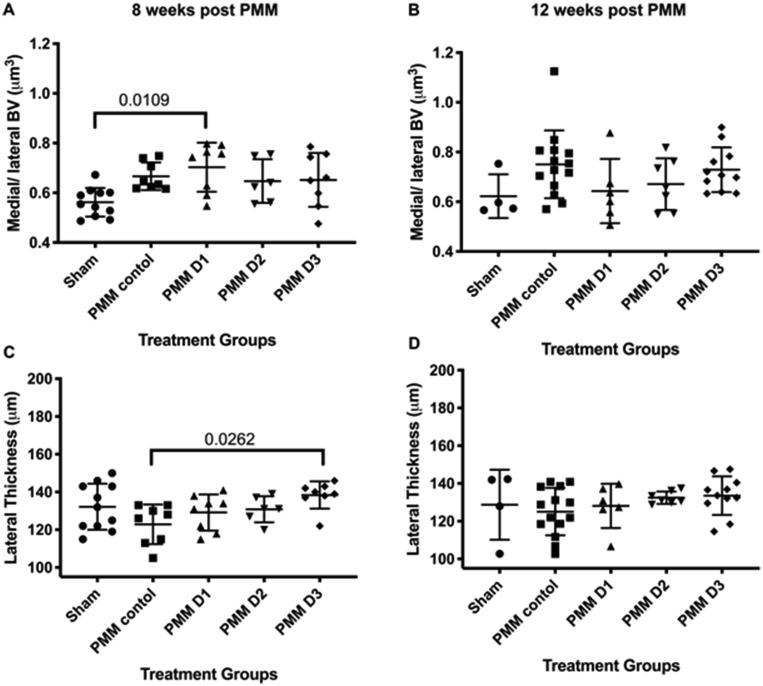
Subchondral bone changes. hUC-MSCs from D1 demonstrated a significantly higher medial/lateral BV than sham mice at 8 weeks (A) but not at 12 weeks (B). When looking at subchondral bone thickness, cells from D3 demonstrated a significantly higher lateral trabecular thickness than the PMM control at 8 weeks (A) but not 12 weeks (D) with no further significant differences observed for any other treatment group or time point. There were no significant differences in any group at 8 or 12 weeks when looking at subchondral bone thickness on the medial side. Data is presented as the mean ± SD. Significance was determined below p < 0.05.

At 8 weeks, administration of cells from D1 demonstrated a significantly higher medial/lateral BV than sham mice (p = 0.0109, Fig. 5A), whilst cells from D3 demonstrated a significantly higher lateral trabecular thickness than PMM control (p = 0.0417, Fig. 5C), with no further significant differences observed for any other treatment group or time point (Fig. 5B and D). Additionally, there were no observed changes in BV/TV throughout the experiment; however, as is normal for C57BL/6 mice (van t’ Hof, unpublished data), the medial side of the joint consistently yielded a slightly higher percentage of bone (BV/TV %) than the lateral side (data not shown).

### 3.3 Histological analyses of joint changes after PMM surgery

**Fig. 6:**
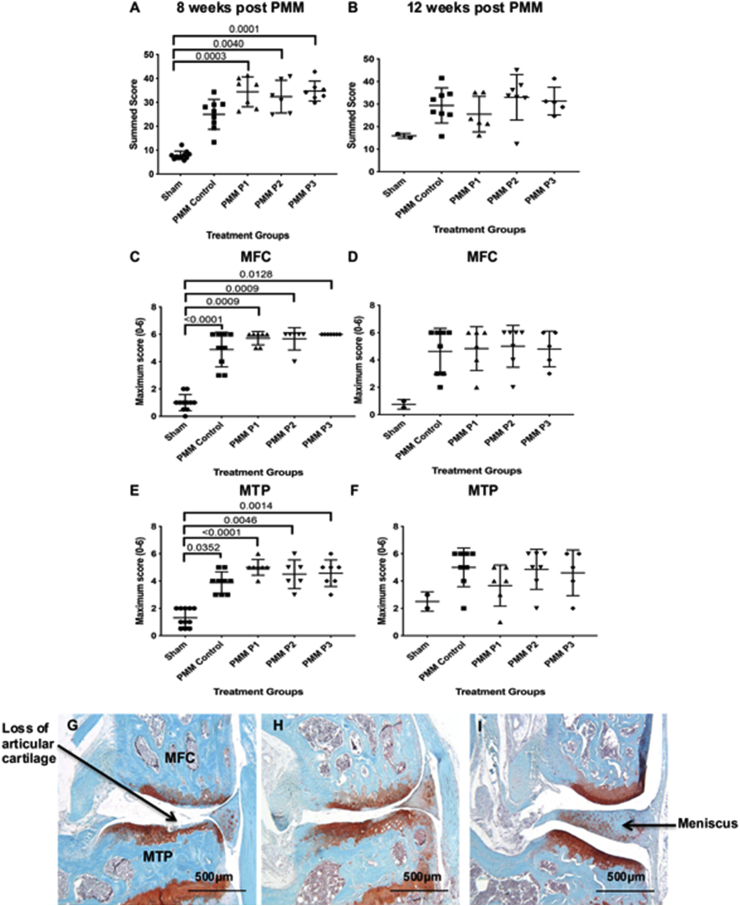
Assessment of cartilage degradation. The summed joint score was significantly higher than the sham for each cell group at 8 weeks (A) but not 12 weeks (B). The maximum scores for the MFC and MTP were also significantly higher than sham and PMM control for each cell group at 8 weeks but not 12 weeks (C–F). Histological changes with extreme loss of GAG (as seen by loss of safranin O staining) and loss of articular cartilage in the mouse knee can be seen in the medial femoral condyle (MFC) and medial tibial plateau (MTP) in the control PMM knee with ‘no cells’ (G) and to a lesser extent in the PMM knee with donor D1 cells (H). No loss of GAG was seen in the sham knee (I). Data is presented as the mean ± SD. Significance was determined below p < 0.05. All sections were stained with safranin O and fast-green counterstain.

**Fig. 7:**
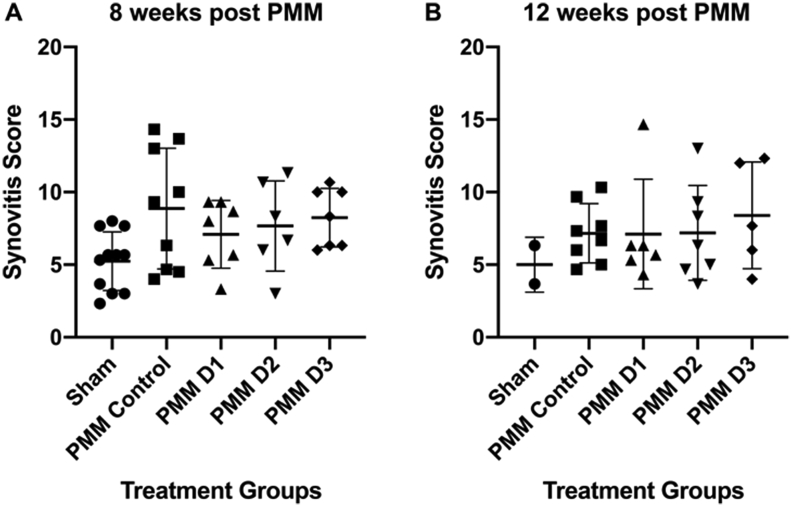
Assessment of synovial reaction: No significant synovitis was detected at either 8 (A) or 12 (B) weeks post-PMM surgery. Data is presented as the mean ± SD.

At 8 weeks but not 12 weeks post-PMM, all groups (±hUC-MSCs) had a significantly higher (worse) summed joint score than the sham control (Figs. 6A–B, G-I). The maximum OARSI scores for the MFC and MTP were also significantly higher for all the PMM groups compared to the sham at 8 weeks but not at 12 weeks (Figs. 6C–F). At neither time-point were there any significant differences on the lateral side between the different groups (data not shown). There were no significant differences in synovitis scores between any of the experimental groups or controls at either 8 or 12 weeks post-PMM (Fig. 7), with no indication of any inflammatory response. There was no evidence of any staining for human emerin in any knee joints, despite positive results for the anti-emerin in human cells in the positive controls (see Supplementary data).

### 3.4 Inflammatory markers

**Fig. 8:**
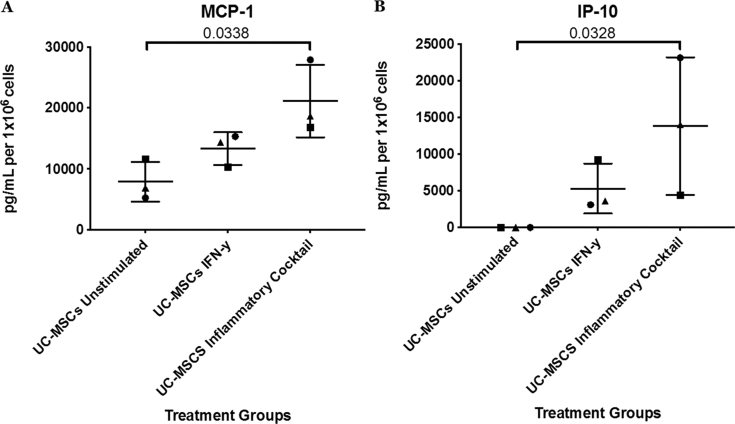
Inflammatory markers. Conditioned media was collected from the following hUC-MSC cell cultures: un-stimulated, stimulated with IFN-γ (25 ng/ml for 24 h) and stimulated with an inflammatory cocktail (25 ng/ml IFN-γ, 50 ng/ml TNF-α 10 ng/ml IL-1β for 24 h) and assessed for the presence of: Monocyte chemoattractant protein 1 (MCP-1; A) and IFN-γ-inducible protein 10 (IP-10; B). MCP-1 and IP-10 were significantly increased following stimulation with an inflammatory cocktail for 24 h, compared with the unstimulated UC-MSCs. Data is presented as the mean ± SD. Significance was determined below p < 0.05. ▪ = donor D1; ▲ = donor D2; ● = donor D3.

All the inflammatory markers in the hUC-MSC conditioned media samples were found to be within the detectable range, but only MCP-1 and IP-10 levels were significantly increased following stimulation with an inflammatory cocktail compared to the unstimulated hUC-MSCs (Fig. 8). In contrast, the levels of inflammatory markers in all murine plasma samples from the 12 week time-point were typically found to be below the lower limit of detection (LLOD) with the exception of MCP-1, VEGF and TNFα but this was not significantly different between groups of mice.

## 4 Discussion

The PMM model as a joint instability model is more severe than the well-established destabilised medial meniscus (DMM) model of OA [24], leading to a faster and more acute onset of OA within 2 weeks compared to 4 weeks in the DMM model [14,15]. Welch et al. (2009) demonstrated that the PMM yielded a greater (worse) OA histological score compared with the DMM model over a 10 week period [15,30]. In our study the total joint OARSI scores were significantly higher in all PMM joints compared with sham joints at both 8 and 12 weeks. This, as well as moderate to severe cartilage damage being observed on the medial side of the joint but less so on the lateral side, is in agreement with previous studies [15,31].

Evidence of synovitis can be observed in 89% of patients with knee OA detected by MRI [32]. Synovitis is increasingly recognised in the pathogenesis of OA, being linked to disease severity in the knee [33]. At both 8 and 12 weeks post-PMM surgery a low level of synovitis persisted but this was not significantly different to mice that received sham surgery. Similar results have been shown in the DMM instability model, with Huesa et al. (2016) observing no differences in synovitis between the DMM and sham group 4 weeks post-surgery [25]. Furthermore, Jackson et al. (2014) suggested that the level of synovitis continually decreases in DMM and sham control groups up to 16 weeks post-surgery [28]*.* Therefore despite utilising a more severe OA model, these results concur with those of others. In addition, the lack of evidence for invasion by inflammatory cells in the synovium or other joint tissues and lack of increased levels of inflammatory markers in any of the plasma samples, suggests that the hUC-MSCs did not evoke an inflammatory response in any of the animals, at least at the time-points measured.

All three populations of hUC-MSCs utilised in this study adhered to the ISCT criteria for MSCs, in terms of all their properties (differentiation potential, plastic adherence and immunoprofile), apart from for the CD14 epitope, which was slightly elevated. We have found previously that CD14 is often elevated on MSCs from bone marrow, synovium and fat pad [34,35]. Following stimulation of hUC-MSCs with IFN-γ and the inflammatory cocktail, our analysis of immunomodulatory gene expression was found to be variable between the 3 donor populations tested. IDO is an immunomodulatory catabolic enzyme responsible for degrading tryptophan in the kynurenine pathway, suppressing T-cells and dampening down an inflammatory response [36]. Our study, as shown previously, demonstrates that under normal culture conditions without inflammatory stimulus, MSCs do not express *IDO*. However, following an inflammatory stimulus such as exposure to IFN-γ, their IDO production is activated [9,37,38]. *TSG6*, which is induced by TNFα, was also upregulated by the hUC-MSCs following stimulation with an inflammatory cocktail. TSG6 is a hyaluronan binding protein and is well known for its potent chondroprotective and anti-inflammatory effects in arthritis [39,40]. The ISCT working proposal also advises that characterisation of stem cells should include activation or ‘licensing’, which involves stimulation with IFN-γ, either alone or with the addition of TNF-α [21]. As we and others have shown previously, the amount of up-regulation of *IDO* in response to an inflammatory stimulus varies between donors, and in this study the cells from donor D1 demonstrated the strongest response to the inflammatory stimuli with the highest up-regulation of *IDO* and *TSG6* [9,38]. This could perhaps be an explanation of the apparent joint protection at 12 weeks post-PMM with regards to joint space narrowing and osteophyte formation in joints treated with this particular population of cells. François et al. (2012) have also suggested that the variation in effectiveness of MSCs reported in clinical trials is likely to be due to intrinsic variability in the immunosuppressive potential of each MSC donor [38]. As far as we are aware, no other *in vivo* studies have characterised this inflammatory response of cells for use in cartilage repair. This novel aspect of this study used UC-MSC donors with vastly different responses to inflammatory cytokines with respect to up-regulation of *IDO* and *TSG6*.

Additionally, it is important to note that the co-stimulatory markers (CD40, 80 and 86) as well as HLA-DR, were not expressed by any of the cell populations prior to, or after stimulation with either IFN-γ alone or the inflammatory cocktail in the present study. The expression of these markers are particularly undesirable on cells destined for allogeneic therapies, as they are all expressed on antigen presenting cells of the immune system, with CD40 also being found on tumour cells [41].

Monocyte chemoattractant protein-1 (MCP-1; also known as CCL2), is a chemokine that was first described for its ability to induce monocyte recruitment, via transendothelial migration, to sites of inflammation by interacting with its receptor, CCR2, found on monocytes. Subsequently MCP-1, has been shown to induce fibroblast proliferation, recruit memory T-lymphocytes and natural killer (NK) cells as well as promote the cellular migration of MSCs [42,43]. Previous work demonstrates that MCP-1 levels in synovial fluid (SF) samples has a moderately positive correlation with radiographic knee OA changes [44] and OA symptomatic severity (as assessed by the Western Ontario and McMaster University Osteoarthritis Index (WOMAC) score) [45]. Furthermore, in the MRL-*lpr* mouse model of arthritis, treatment with an MCP-1 antagonist, MCP-1 (9–76), prior to disease onset greatly reduced OA progression whereas native MCP-1 enhanced the onset and aggravated joint inflammation [46]. In our study, following stimulation with an inflammatory cocktail the levels of MCP-1 were significantly increased in hUC-MSCs (particularly those from donors 2 and 3 (D2 and D3)) compared with the un-stimulated hUC-MSCs. This could potentially explain the slight joint protection seen in mice receiving cells from donor D1, with regards to joint space narrowing, with these cells not significantly increasing MCP-1 expression.

Interferon-y-inducible protein 10 (IP-10, also called CXCL10) is an inflammatory chemokine inducible by IFN-γ and TNFα. Following stimulation with IFN-γ we saw a large increase in IP-10 levels and even more so with the combination of IL-1β, IFN-γ and TNFα, particularly for cells from donors 2 and 3 (D2 and D3). Previous work demonstrated that IP-10 is increased in numerous arthritic diseases, including rheumatoid arthritis, and can hone leukocytes to inflamed tissues [47]. IP-10 can also promote osteoclastogenesis, by inducing RANKL in activated CD4^+^ T-cells [48] leading to the erosion of bone and exacerbation of inflammation. When selecting and banking MSCs for allogeneic therapies, it is important to determine their response to inflammatory stimuli, as lower production of MCP-1 and IP-10 may be more desirable for the treatment of arthritic patients and other inflammatory diseases [47,48].

Donor-donor variation is not uncommon and the varying degrees of immunosuppressive potential could be due to an intrinsic disparity in sensitivity and plasticity of the cells to inflammatory cytokines [38]. This stresses the importance of cell characterisation when creating cell banks for allogeneic therapies. Should cells be transplanted into an inflammatory environment, or if the primary mechanism of therapeutic action is immunomodulation, then understanding the immunomodulatory potential is vital.

Pre-clinical OA models are helpful for predicting treatment response and other outcome measures. However, no disease model has the capacity to completely and accurately mimic the human condition with regards to pathogenic mechanisms, treatment methods and responses. For example, when a human patient presents in the clinic with degeneration resulting in/from malalignment or a destabilised knee joint, an orthopaedic surgeon is likely to stabilise the joint (e.g. through an osteotomy, or meniscal repair/transplantation) prior to or whilst simultaneously performing cell therapy. In contrast, in our study, hUC-MSCs were injected into unstable joints with a greater degree of disease severity, more akin to end-stage OA in the human. Perhaps the lack of improvement in joint health with all cell populations used indicates that cell therapy is inappropriate for end stage OA, at least for reversing major structural changes.

While it is evident from our study that the injected hUC-MSCs may not be able to ameloriate osteophyte formation, or prevent complete cartilage damage following a PMM, they do not appear to advance the pathogenesis of the disease. This supports the hypothesis that a mechanical instability model such as the PMM may be too severe to overcome [49]. Therefore, it may be appropriate to determine whether hUC-MSCs can improve osteochondral defects that lead to secondary OA, or use a milder OA pre-clinical model to determine their effectiveness as a cell therapy.

## 5 Study limitations

Limitations of the model include the late stage time course, speed of disease onset and the fact that blood was only collected at the 12 week time-point. In addition and as in many other studies, only male mice were used in this study as they typically develop more severe cartilage degradation when compared with females, in both spontaneous and surgically induced OA models [50]. A further possible limitation is that pain was not assessed in this study.

## 6 Conclusion

In conclusion, our results demonstrate that although the transplanted hUC-MSCs did not all recover joint damage induced by the PMM as assessed histologically, the implanted cells did not appear to elicit an inflammatory response in the treated mice at the time-points studied here. Furthermore, there was marked variability between the UC donors, with significant reduced loss of joint space at 12 weeks being seen with one donor's cells. This suggests that some donors' cells may have a greater therapeutic potential than others, highlighting the importance of donor cell characterisation for allogeneic cell therapies. Of course, the use of a less severe OA model might be more appropriate than the model of severe OA as used here, thus enabling a greater insight into the effectiveness of hUC-MSCs in the prevention or delay of the development of this disease, at least in the murine knee joint.

## Author contributions

SR, CM, GBG, and RvTH conceived of and designed the study. GBG and PM provided the study subjects, RvTH the micro-CT expertise and CM the hUC-MSCs. JP, CM, HM, SR, GBG and RvTH collected the data. JP analysed the data and JP, CM, HM, GM, RvTH, GBG and SR assisted in interpreting it. JP, CM, HM, GBG, RvTH, PM and SR drafted and critically revised the manuscript for important intellectual content. SR obtained funding for the study. All authors read and approved the final manuscript. SR (sally.roberts4@nhs.net) takes responsibility for the integrity of the work as a whole, from inception to the finished manuscript.

## Role of funding source

10.13039/501100012041Versus Arthritis supported salaries and consumables for CM, HM and JP via grants 19429, 20815 and 21122 and the 10.13039/501100000265Medical Research Council (grant number MR/L010453/1) supports HM's salary. The sponsors had no involvement in the study design, data collection and interpretation, or preparation of the manuscript.

## Declaration of Competing Interest

None of the authors have any conflict of interest.
