# Supplementary material for: Injected human umbilical cord-derived mesenchymal stromal cells do not appear to elicit an inflammatory response in a murine model of osteoarthritis
Source: Osteoarthr Cartil Open. 2020 Feb 19;2(2):100044. doi: 10.1016/j.ocarto.2020.100044 (PMC7307639; doi:10.1016/j.ocarto.2020.100044)

| Score | Pannus | Synovial Membrane (Hyperplasia Severity) | Sub-synovial stroma | Sub-synovial Inflammation | Synovial Exudate |
| --- | --- | --- | --- | --- | --- |
| Description | Defined as fibrous tissue/synovium/ inflammatory cell outgrowth spreading over the surface of the bone and/or cartilage at the joint margins. | Score this superior to the meniscal remnant. Do not score the cells actually attached to the tibia or femur or cells on the surface of the meniscus itself and avoid peri-meniscal plica. Score the maximum hyperplasia seen anywhere along this area. | Score the stroma adjacent to the synovial membrane. | Score this superior to the meniscal remnant. The infiltration of inflammatory cells (neutrophils, macrophages and/or lymphocytes) is evaluated. | Infiltration of inflammatory cells (neutrophils, macrophages and/or lymphocytes) in the synovial cavity. |
| 0 | No Pannus | 1 cell thick | Synovial stroma shows normal cellularity. | No inflammatory cells | No inflammatory cells or fibrin in the synovial cavity. |
| 1 | Mild: Pannus has migrated on bone but not encroaching on cartilage. | Mild: 2-3 cells thick | The cellularity is slightly increased. | Occasional scattered inflammatory cells – or perivascular. | Inflammatory cells and/or fibrin colt in the synovial cavity – may be restricted to recesses. |
| 2 | Moderate: Pannus has migrated < 1x cartilage depth. | Moderate: 4-5 cells thick | The cellularity is moderately increased, multinucleated cells might occur. | Focal areas of dense subsynovial WBC infiltrate – but still predominantly normal subsynovial areolar connective tissue present. |  |
| 3 | Severe: Pannus has migrated > 1x cartilage depth. | Severe: > 6 cells thick | The cellularity is greatly increased, multinucleated giant cells, pannus formation and rheumatoid granulomas might occur. | Widespread dense subsynovial WBC infiltrate – markedly reduced or little/no normal areolar connective tissue evident or lymphoid follicle formation. |  |

*Table 1. Criteria for the grading of synovitis. Modified from Jackson et al* and Krenn *et al* (Krenn *et al.*, 2006; Jackson *et al.*, 2014)*.*

| Human CM | Mouse Plasma PMM |
| --- | --- |
| Granulocyte-macrophage colony-stimulating factor (GM-CSF) | GM-CSF |
| Interleukin-1 receptor antagonist (IL-1RA) | IL-1β |
| IL-4 | IL-4 |
| IL-6 | IL-6 |
| IL-8 | IL-10 |
| IL-10 | Tumor necrosis factor alpha (TNFα) |
| IFN-γ-inducible protein 10 (IP-10) | Interferon gamma (IFNγ) |
| Monocyte chemoattractant protein 1 (MCP-1) | MCP-1 |
| Vascular endothelial growth factor (VEGF) | VEGF |
| Stromal cell-derived factor 1 (SDF-1α) | Transforming growth factor beta-3 (TGFβ-3) |

*Table 2. MSD analytes observed in the human UC conditioned medium (CM) and mouse plasma post-PMM.*

Figure 1. Emerin identification of human UC cells. Emerin was clearly detected (green staining) in human umbilical cord tissue (A) and an unoperated murine knee joint that received hUC-MSCs (B). Positive DAPI staining (blue) for cell nuclei was clearly present in cells in all tissue sections (A-E). Despite receiving hUC-MSCs, emerin staining was not detectable in samples at the 8 week time-point (C). All IgG control sections were negative, apart from for DAPI staining.


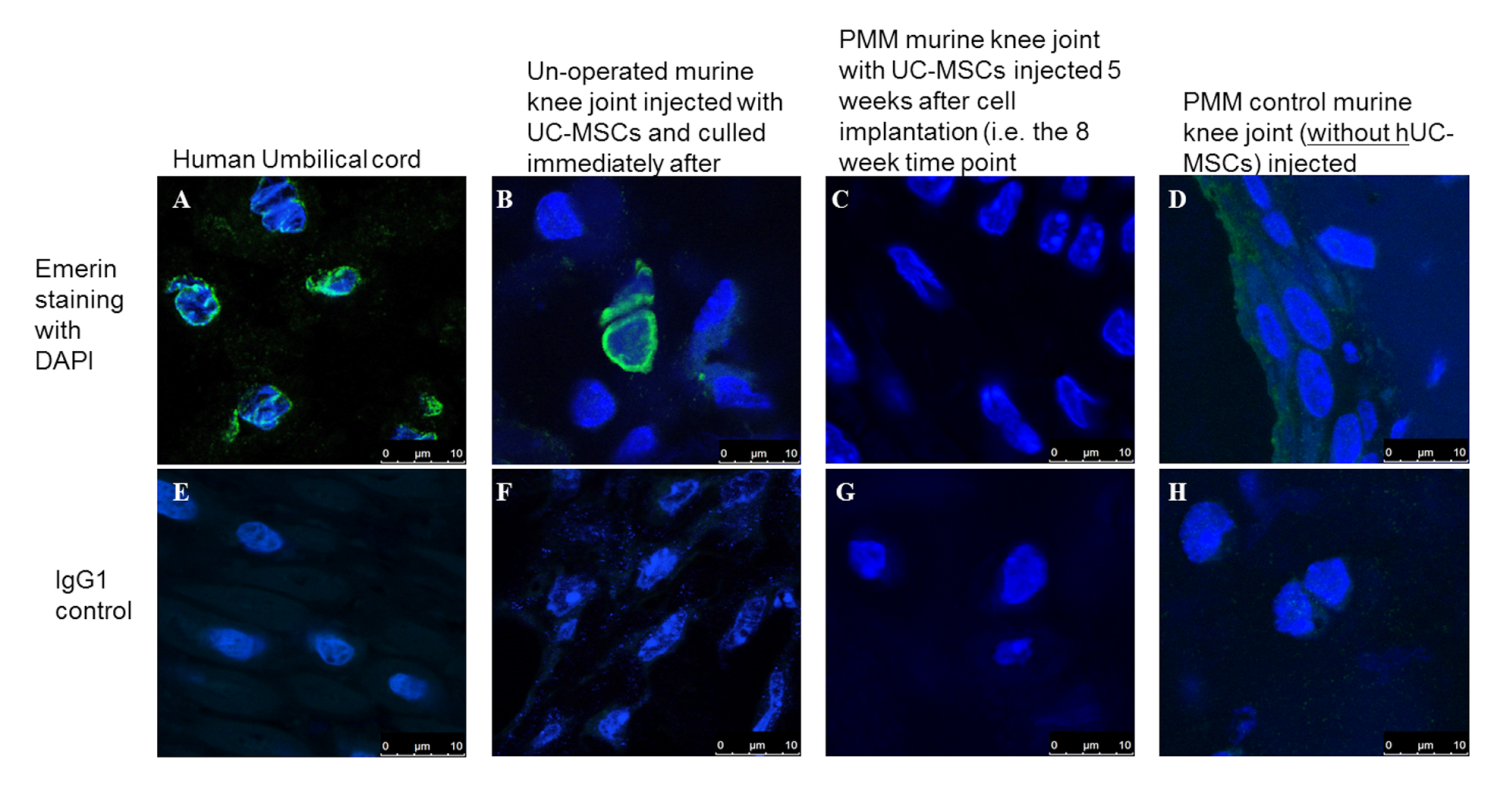

Supplement: Multimedia component 1 [file mmc1.docx]
